# The potential performance of serum albumin to globulin ratio, albumin and globulin in the diagnosis of periprosthetic joint infection and prediction of reinfection following reimplantation

**DOI:** 10.1186/s12891-022-05533-0

**Published:** 2022-07-30

**Authors:** Haitao Zhang, Shuihua Xie, YiJin Li, Jiahao Li, Peng Deng, Huiliang Zeng, Houran Cao, Min Liu, Jie Li, Wenjun Feng, Pengcheng Ye, Yingjie Ge, Jianchun Zeng, Yirong Zeng

**Affiliations:** 1 The First Clinical of Medical School, Guangzhou University of Chinese Medicine, NO.12 Jichang Road, District Baiyun, Guangzhou, 510405 Guangdong China; 2 The First Department of Orthopedics, Jiangxi Province Hospital of Integrated Chinese and Western Medicine, NO.90 Bayi Road, District Xihu, Nanchang, 330003 Jiangxi China; 3 Department of Orthopaedics, The First Affiliated Hospital of Guangzhou University of Chinese Medicine, NO.16 Jichang Road, District Baiyun, Guangzhou, 510405 Guangdong China; 4 The Tenth Department of Orthopedics, Foshan Hospital of Chinese Medicine, NO.6 Qinren Road, District Chancheng, Foshan, 528000 Guangdong China; 5 The Director of the Orthopedic Department of, Guangdong Province Hospital of Traditional Chinese Medicine, NO.111 Dade Road, District Yuexiu, Guangzhou, 510000 Guangdong China; 6 The Affiliated Hospital of Jiangxi University of Traditional Chinese Medicine, No.445 Bayi Avenue, Nanchang, 330000 Jiangxi China

**Keywords:** Periprosthetic joint infection, Performance, Diagnosis, Serum albumin to globulin ratio, Reimplantation

## Abstract

**Background:**

This study was conducted to evaluate the performance of serum albumin (ALB), globulin (GLO), and albumin to globulin ratio (AGR) in the diagnosis of PJI and prediction of reinfection following reimplantation in PJI patients who underwent two-stage revision.

**Methods:**

We perform a retrospective data collection on identified patients who underwent revision arthroplasties in our institution from January 2010 to January 2020. A total of 241 patients were stratified into: group A (PJI), group B (aseptic loosening). Fifty-five patients who underwent two-stage revision in group A were assigned to group C. Group C was stratified into subgroup 1 (reinfection) and subgroup 2 (non-reinfection). Receiver operating characteristic curves were used to evaluate the utility of serum markers for diagnosing PJI and predicting reinfection following reimplantation.

**Results:**

In the diagnosis of PJI, there were significant differences in the levels of ALB, GLO, and AGR between groups A and group B (*P* < 0.05). The AUC value of serum AGR (0.851) was similar to ESR (0.841) and CRP (0.866) (all *p* > 0.05). The AUC values of serum ALB and GLO were 0.757 and 0.753, respectively. As for predicting reinfection following reimplantation, the serum ALB in the non-reinfection group was higher than that in the reinfection group (*p* = 0.041). The AUC value of serum ALB was 0.7.

**Conclusion:**

AGR was promising adjunct marker for the diagnosis of PJI, similar to CRP and ESR. ALB and GLO have an acceptable value for the diagnosis of PJI. ALB may be expected to be a kind of effective marker for predicting reinfection following reimplantation.

## Background

The incidence of periprosthetic joint infection (PJI) after total joint replacement (TJA) is 1–3% [1–3], while the risk of reinfection after PJI revision increases by almost tenfold [4]. Early and precise distinction between PJI and aseptic loosening plays a vital role in subsequent treatment. In recent decades, although the diagnosis of PJI has been developed to a certain extent, including the emergence of the American Academy of Orthopedic Surgeon (AAOS)'s guidelines, Musculoskeletal Infection Society (MSIS) criterion, and a large number of burgeoning biomarkers, the diagnosis of PJI and the determination of the timing before reimplantation remain challenging due to the lack of an exclusive standard of 100% diagnostic performance [5, 6]. In addition, the ever-changing infectious bacterial spectrum is a momentous factor in the difficulty of PJI diagnosis. Based on this context, it is necessary to invest considerable effort in exploring novel markers to improve the diagnosis of PJI.

Albumin (ALB) and globulin (GLO) are indispensable components in serum protein [7, 8]. An early study reported that the level of albumin in chronic inflammatory diseases shows a downward trend, while an increase in GLO levels can reflect an increase in proinflammatory cytokines, which has been shown to be an important marker of chronic inflammation [9]. Basic studies have explained that the main mechanism of decreased ALB production and elevated GLO levels during chronic inflammation or infection may be the increase in the fractional catabolic rate and the accumulation of inflammatory cytokines in serum GLO. Recently, an increasing number of studies have demonstrated that serum GLO and the albumin to globulin ratio (AGR) present regularly vary in patients with infection, hepatitis, or tumors, indicating that they play a significant role in the immune and inflammatory systems [10–12]. Hence, they are clinically regarded as non-invasive markers to reflect the inflammatory level of patients and diagnose tumors. The above findings establish a theoretical foundation for the use of serum ALB, GLO, and AGR in the diagnosis of PJI. A previous study has illustrated that serum GLO and AGR are auxiliary biomarkers for the diagnosis of PJI [13]. Nevertheless, the small size of the PJI group in that study might compromise its clinical practice. Besides, few studies have explored the value of serum ALB, GLO, and AGR to predict reinfection following reimplantation in patients with two-stage revision.

As a consequence, we attempt to evaluate (1) the performance of serum ALB, GLO, and AGR compared with conventional biomarkers serum white blood cell count (WBC), erythrocyte sedimentation rate (ESR), and C-reactive protein (CRP) in the diagnosis of PJI and (2) whether serum ALB, GLO, and AGR can be used to predict reinfection following reimplantation in PJI patients who underwent two-stage revision.

## Methods

### Study design, inclusion, and exclusion criteria

This study was approved by the institutional review board. We perform a retrospective data collection on identified patients who underwent revision arthroplasties in our institution from January 2010 to January 2020. The process of data collection was consecutive and nonselective. A total of 306 patients were initially included. We excluded liver and kidney dysfunction (*n* = 12), hypogammaglobulinemia (*n* = 1), and periprosthetic fracture (*n* = 9) missing data (*n* = 3). Additionally, patients with urinary tract infection (*n* = 1), malignancy (*n* = 11), rheumatoid arthritis (*n* = 13), ankylosing spondylitis (*n* = 10), or acute infection (*n* = 4) were also excluded from the analysis. Acute infection was determined by the occurrence of PJI symptoms within 6 weeks after arthroplasty [14]. It's worth explaining that there are some possible reasons why there were only 4 acute PJI. On the one hand, the time of diagnosis criteria is not uniform, on the other hand, patients with acute infection after primary TJA in our institution might also go to other hospitals. Besides, a few patients with acute PJI develop chronic PJI as a result of antibiotic use. The patients with PJI and aseptic loosening were distinguished according to the Musculoskeletal Infection Society (MSIS) criteria in 2013 (Table 1) [15]. Thus, the 241 patients included in this study were stratified into Group A (PJI) and Group B (aseptic loosening). Furthermore, fifty-five patients who underwent two-stage revision in Group A were assigned to Group C, and the remaining patients with non-two-stage revision were not analyzed further. Patients in Group C were followed up for at least 12 months to determine whether reinfection had occurred. Infection eradication was also assessed based on the 2013 version of the MSIS criteria [15]. Patients who discontinued reimplantation and underwent additional spacer replacement due to suspected infection were considered to have uneradicated infection. Subsequently, Group C was stratified into subgroup 1 (reinfection) and subgroup 2 (non-reinfection).

**Table 1:** The Musculoskeletal Infection Society Criteria issued in 2013

Two Criteria	Specific Item
Major Criteria (Met one of the major criteria)	a. Two positive periprosthetic tissue cultures with phenotypically identical organisms
	b. A sinus tract communicating with the joint
Minor criteria (Met any three of the minor criteria)	a. Elevated serum CRP > 10 mg/L AND ESR > 30 mm/h
	b. Elevated synovial fluid white blood cell count > 3000cells/ml OR change on leukocyte esterase test strip (+ or + +)
	c. Elevated synovial fluid polymorphonuclear neutrophil percentage > 80%
	d. Positive histological analysis of periprosthetic tissue (> 5 neutrophils per high-power field in 5 high-power fields (× 400))
	e. A single positive culture

*PJI* Periprosthetic joint infection, *ESR* Erythrocyte sedimentation rate, *CRP* C-reactive protein, *SF* synovial fluid, *WBC* White blood cell, and *PMN%* Polymorphonuclear neutrophil percentage

PJI is diagnosed when one of the major criteria presents, or three of five of the minor criteria present

### Data recording

The baseline characteristic information (sex, age, body mass index, involved joint), clinical symptoms, diagnosis, previous history, and preoperative laboratory serum biomarker levels (WBC, ESR, CRP, ALB, GLO, AGR) of patients were recorded from the electronic medical record database. It should be explained that the serum biomarker collection in Group A and Group B was within one week prior to revision surgery, while the serum biomarker collection in Group C (including subgroup 1 and subgroup 2) was within one week prior to reimplantation or additional spacer exchange. The data levels of patients in Group A and Group B were compared to determine the performance of biomarkers in the diagnosis of PJI. The data levels of subgroup 1 and subgroup 2 were compared to evaluate the utility of biomarkers in predicting reinfection following reimplantation. In our institution, fasting blood samples were routinely collected in the morning of the second day after admission and sent by nurses to the laboratory as soon as possible for testing. The pathogens of 89 PJI patients and 11 patients with persistent or new infections after two-stage revision are summarized in detail.

### Statistical analysis

All data analysis and image production were performed by IBM SPSS Statistics version 23 (IBM, Armonk, New York), Med Calc for Windows version 18.2.1.0 (Med Calc Software by, Ostend, Belgium), and GraphPad Prism version 8. All quantitative data are presented as the mean ± standard deviation. The Mann–Whitney test and t test were used to assess the quantitative data, and the chi-square test was used to compare the categorical variables. A *p* value < 0.05 was considered statistically significant. Receiver operating characteristic (ROC) curves were drawn, and the area under the curve (AUC), sensitivity, specificity, positive likelihood ratio (PLR), and negative likelihood ratio (NLR) were calculated to evaluate the performance of WBC, ESR, CRP, ALB, GLO, and AGR in the diagnosis of PJI and the prediction of reinfection following reimplantation. The optimal threshold range of the above biomarkers was determined according to the Youden J index. Diagnostic performance was measured according to the criteria of a previous study: an AUC value greater than 0.9 indicated fairly good accuracy, 0.8 to 0.9 indicated good accuracy, 0.7 to 0.8 indicated acceptable accuracy, and below 0.7 indicated poor accuracy [16].

## Results

### Patient demographics

**Fig. 1:**
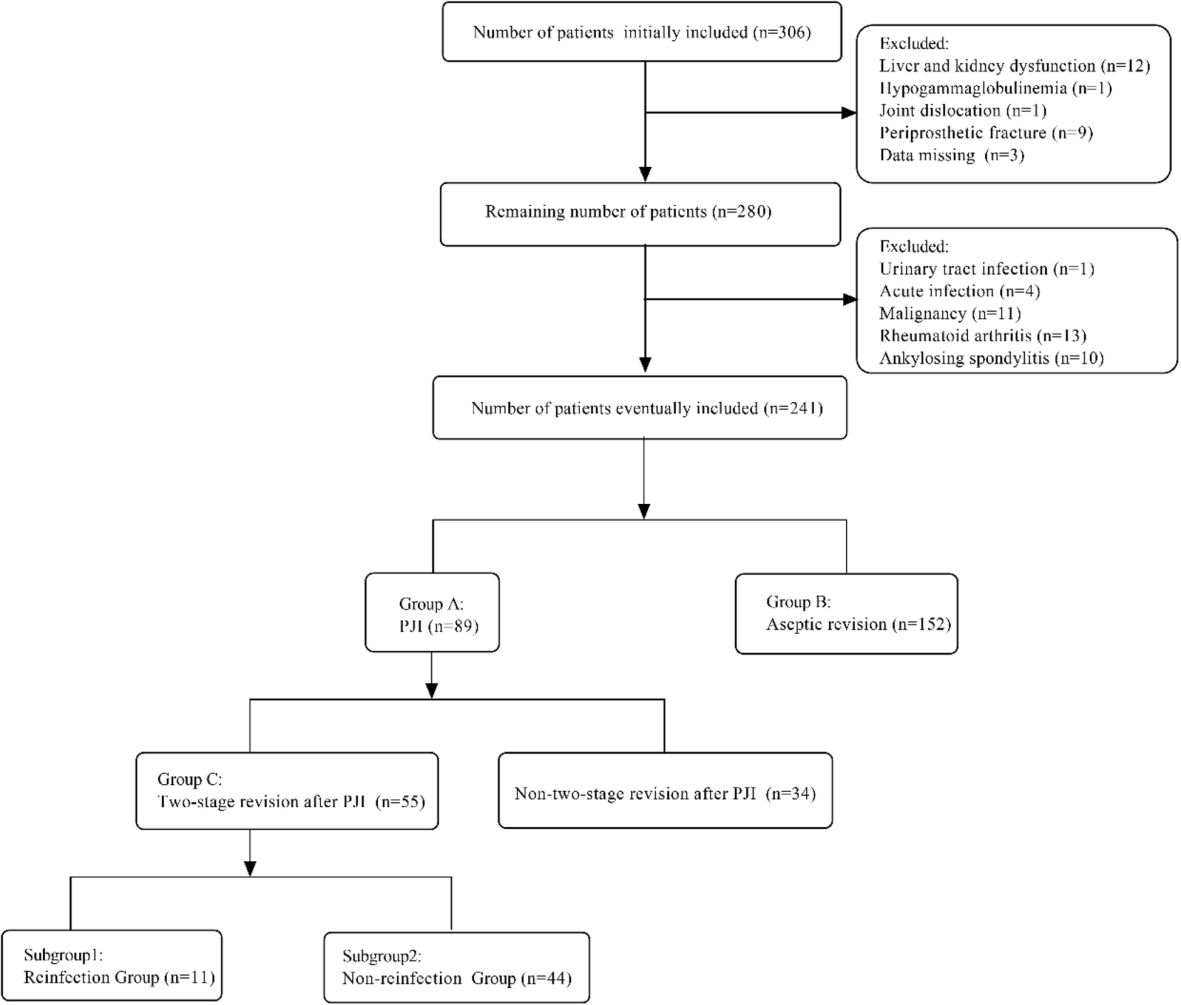
Flowchart of case screening and grouping

A total of 241 patients were included in our study, including Group A (*n* = 89) and Group B (*n* = 152). Moreover, fifty-five patients who underwent two-stage revision in Group A were assigned to Group C. Group C consisted of 11 reinfected (subgroup 1) and 44 non-reinfected (subgroup 2) patients. The case screening process is shown in Fig. 1. The characteristics of the patients are shown in Table 2. There was no significant difference in other characteristics between Group A and Group B except for the joint location (*P* < 0.001). Furthermore, all baseline characteristics between subgroups 1 and 2 were not statistically significant. The mean follow-up time of Group C was 43.34 ± 31.62 months. Follow-up results showed that 11 patients (20%) experienced persistent infection or new infections, including 4 patients who underwent additional spacer insertion and 7 patients who underwent reimplantation of the prosthesis.

**Table 2:** Demographics of patients

	Group A (*N* = 89)	Group B (*N* = 152)	*P* value (Group A vs B)	Group C (*N* = 55)		*P* value (Subgroup1 VS 2)
				Subgroup 1(*N* = 11)	Subgroup 2(*N* = 44)	
Sex			0.826			1.000
Male	37	61		4	16	
Female	52	91		7	27	
Age (y)						
61.46 ± 13.70		65.11 ± 9.69	0.147	61.63 ± 11.19	60.32 ± 13.13	0.761
BMI (kg/m ^2^)						
23.83 ± 4.24		23.81 ± 3.43	0.955	25.24 ± 3.32	23.66 ± 4.09	0.740
Joint						
			**0.001**			0.194
Hip	57	130		4	28	
Knee	32	22		7	16	

Group A, Periprosthetic Joint Infection; Group B, aseptic loosening. The 55 patients in group C were patients in group A who underwent two-stage revision. Subgroup 1, reinfection after two-stage revision; Subgroup 2, non-reinfection after two-stage revision. Bold numbers indicate a statistical difference

### Diagnostic performance of serum biomarkers for PJI

**Fig. 2:**
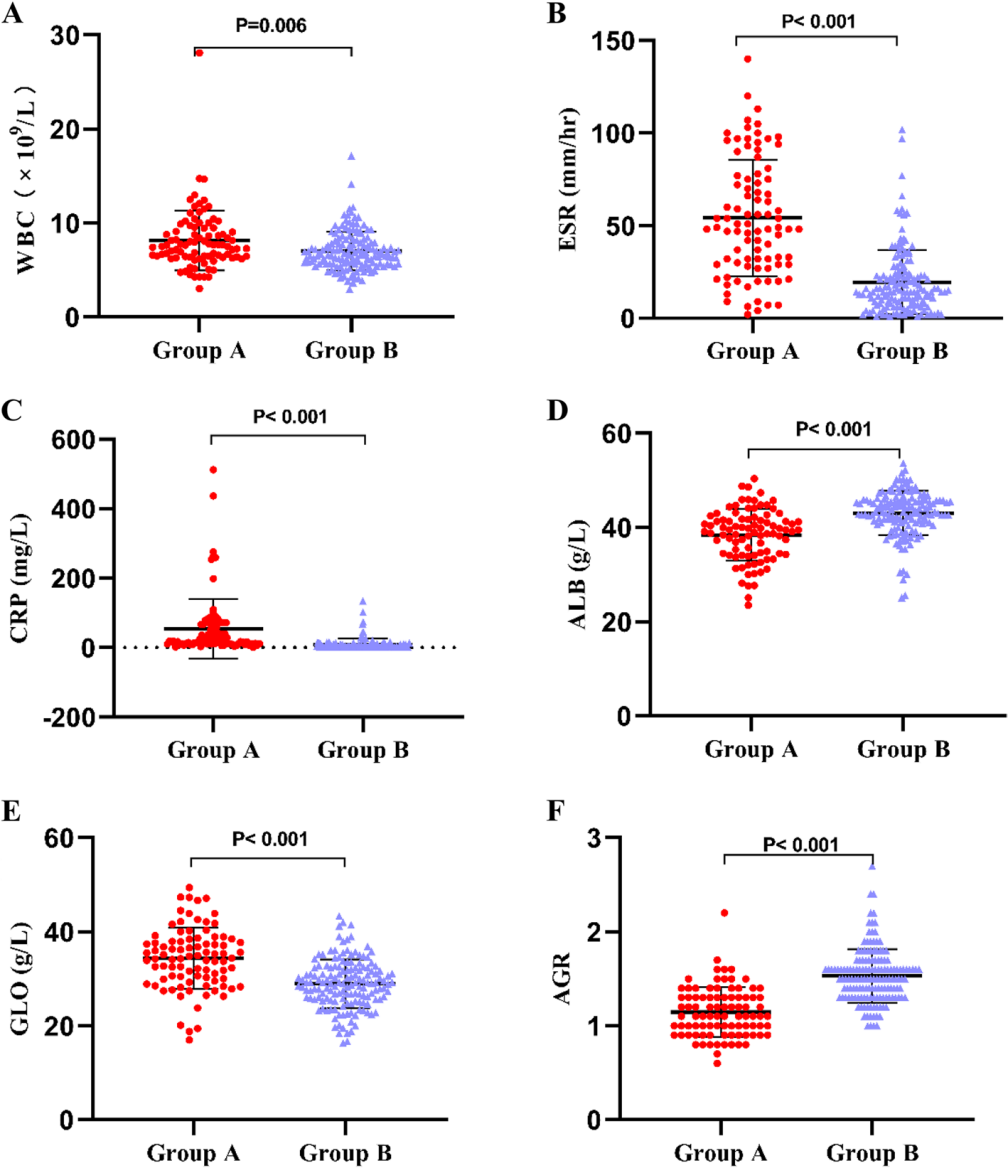
Comparison of Serum WBC, ESR, CRP, ALB, GLO and AGR between group **A** (PJI) and group **B** (Aseptic loosening)

The average levels of serum WBC, ESR, CRP, and GLO in Group A were significantly higher than those in Group B (all *P* < 0.05). In contrast, the average levels of serum ALB and AGR in Group A were significantly lower than those in Group B (all *P* < 0.001), as shown in Table 3 and Fig. 2.

**Table 3:** Comparison Biomarker Levels in the 3 Groups

Markers	Group A	Group B	*P* Value
WBC (× 10^9^/L)	8.12 ± 3.19	7.05 ± 2.04	**0.006**
ESR (mm/hr)	54.05 ± 31.38	19.42 ± 17.26	** < 0.001**
CRP (mg/L)	54.69 ± 86.40	9.15 ± 16.73	** < 0.001**
ALB (g/L)	38.44 ± 5.43	43.07 ± 4.70	** < 0.001**
GLO (g/L)	34.33 ± 6.49	28.93 ± 5.16	** < 0.001**
AGR	1.15 ± 0.26	1.53 ± 0.29	** < 0.001**

Group A, Periprosthetic Joint Infection; Group B, aseptic loosening. *WBC* White blood cell count, *ESR* Erythrocyte sedimentation rate, *CRP* C-reactive protein, *ALB* Albumin, *GLO* Globulin, *AGR* Albumin to globulin ratio. *P* value was calculated by the Mann–Whitney U test. Bold numbers indicate a statistical difference

**Fig. 3:**
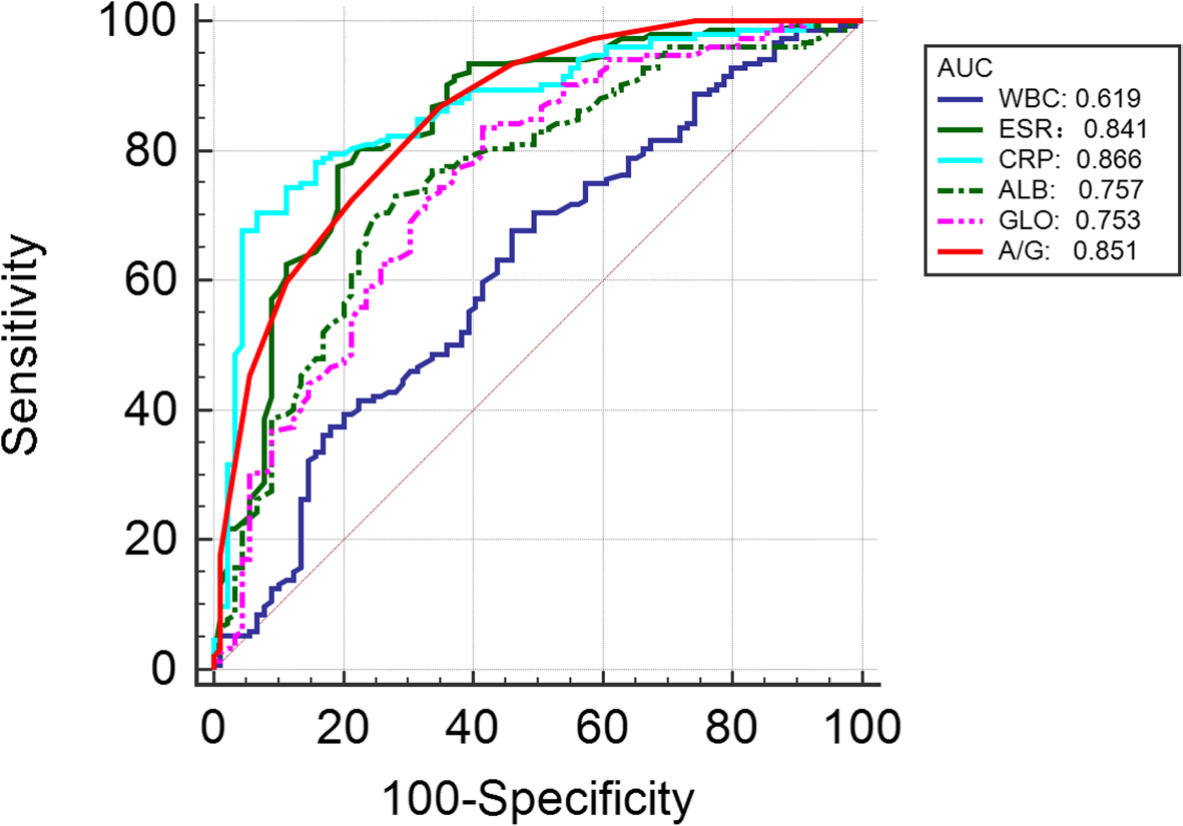
ROC curve of serum WBC, ESR, CRP, ALB, GLO and AGR in the diagnosis of PJI

The performance of serum biomarkers in the diagnosis of PJI is shown in Table 4 and Fig. 3. The AUC values of the conventional markers WBC, ESR, and CRP for the diagnosis of PJI were 0.619, 0.841, and 0.866, respectively. In comparison, the AUC values of the novel markers ALB, GLO, and AGR for the diagnosis of PJI were 0.757, 0.753, and 0.851, respectively. The AUC of AGR was similar to that of ESR and CRP (all *P* > 0.05). The optimal thresholds (sensitivity, specificity) of WBC, ESR and CRP were 7.38 × 109/L (53.93%, 67.76%), 25 mm/hr (80.90%, 77.63%), and 6.63 mg/L (93.26%, 70.39%), respectively. The optimal thresholds (sensitivity, specificity) for ALB, GLO, and AGR were 41.7 g/L (75.28%, 69.74%), 33.6 g/L (58.43%, 83.55%), and 1.2 g/L (65.17%, 86.84%), respectively. The AUC value of ESR combined with CRP was 0.869. The AUC value of ESR, CRP combined with AGR was 0.891. The AUC value of ALB, GLO combined with AGR was 0.856.

**Table 4:** The performance of single and combinative serum markers for the diagnosis of PJI

Serum Markers	Sensitivity (%)	Specificity (%)	PPV (%)	NPV (%)	AUC (95% CI)	Threshold	Youden Index
WBC	53.93	67.76	49.48	71.53	0.619 (0.555 to 0.681)	7.38 × 10^9^/L	0.217
ESR	80.90	77.63	67.92	87.41	0.841 (0.789 to 0.885)	25 mm/hr	0.585
CRP	93.26	70.39	64.84	94.69	0.866 (0.817 to 0.907)	6.63 mg/L	0.637
ALB	75.28	69.74	59.29	82.81	0.757 (0.698 to 0.810)	41.7 g/L	0.450
GLO	58.43	83.55	67.53	77.44	0.753 (0.693 to 0.806)	33.6 g/L	0.419
AGR	65.17	86.84	74.36	80.98	0.851 (0.800 to 0.894)	1.2	0.520
ESR + CRP	85.39	78.95	70.37	90.22	0.869(0.820 to 0.909)	-	-
					0.856(0.805 to 0.898)		
					0.891(0.844 to 0.927)		
AGR + ALB +	69.66	90.13	92.33	63.49		-	-
GLO							
AGR + ESR +	78.65	86.18	76.92	87.33		-	-
CRP							

*PJI* Periprosthetic joint infection, *ROC* Receiver operating characteristic, *PPV* Positive predictive value, *NPV* Negative predictive value, *AUC* Area under the curve, *WBC* White blood cell count, *ESR* erythrocyte sedimentation rate, *CRP* C-reactive protein, *ALB* Albumin, *GLO* Globulin, *AGR* Albumin to globulin ratio

### The prediction performance of serum biomarkers for reinfection following reimplantation

**Fig. 4:**
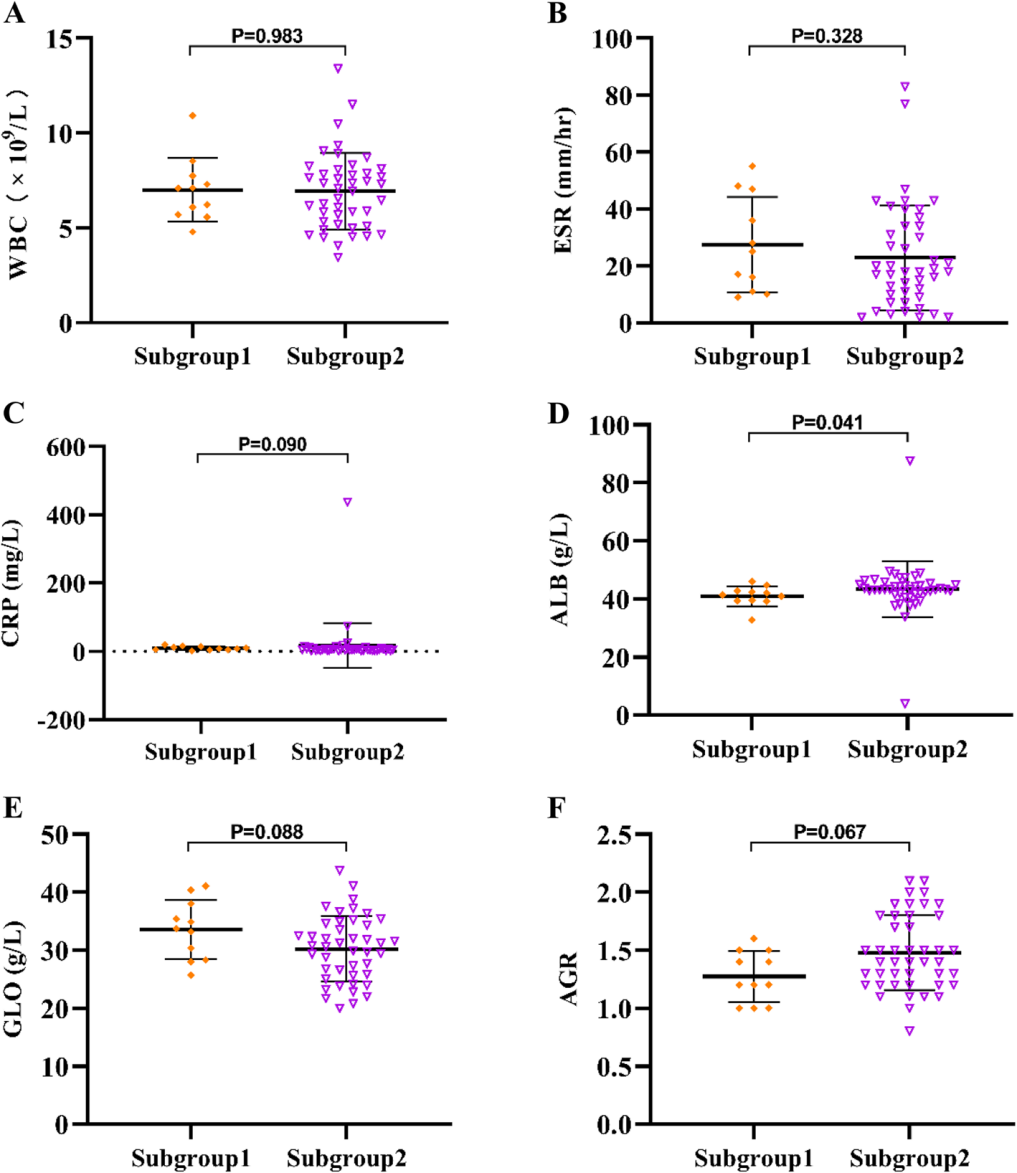
Comparison of Serum WBC, ESR, CRP, ALB, GLO and AGR between Subgroup 1 (reinfection) and Subgroup 2(non-reinfection)

**Fig. 5:**
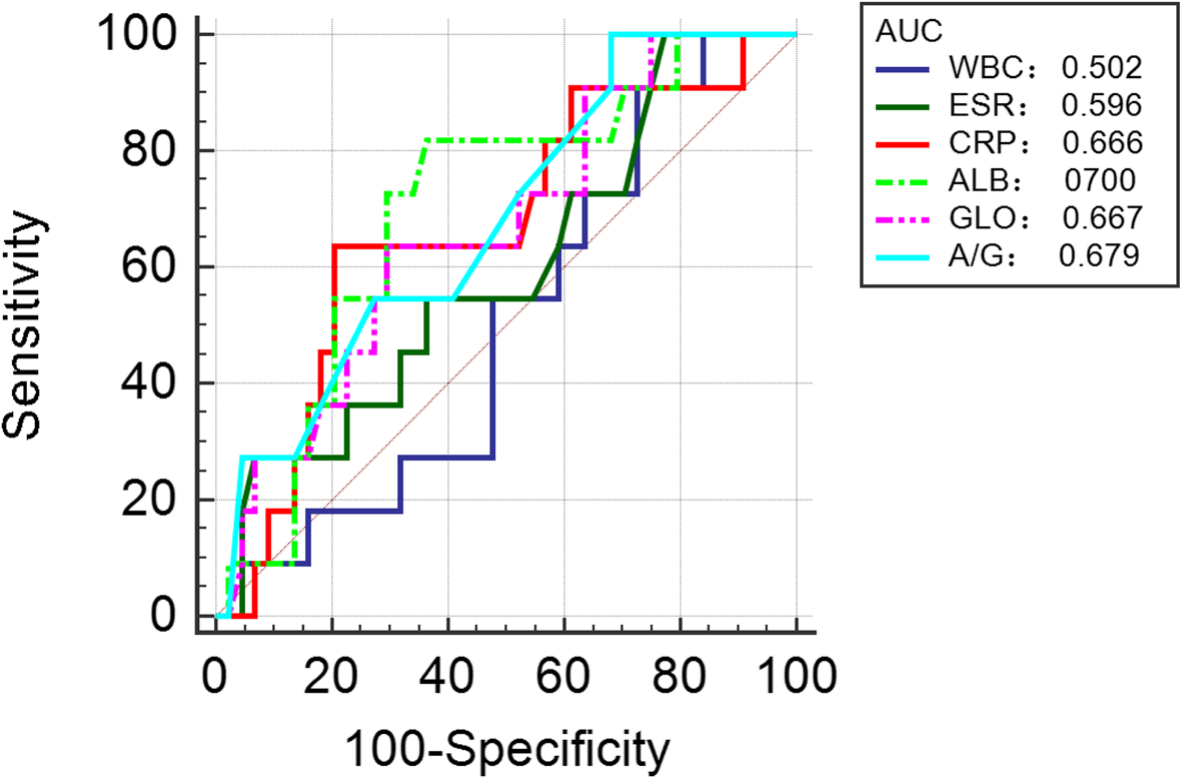
ROC Curve for serum WBC, ESR, CRP, ALB, GLO and AGR in predicting reinfection following reimplantation

A comparison of serum marker levels between subgroup 1 and subgroup 2 patients before reimplantation is shown in Table 5 and Fig. 4. The performance of serum biomarkers in predicting reinfection following reimplantation is demonstrated in Table 6 and Fig. 5. The results showed that the AUC values of WBC, ESR, CRP, GLO, and AGR were all below 0.7, which were 0.502, 0.596, 0.666, 0.667, and 0.679, respectively. ALB had the optimum AUC (0.700). In addition, the optimal threshold (sensitivity, specificity) of ALB was 42.7 g/L (81.82%, 63.64%). The AUC value of ESR combined with CRP was 0.605. The AUC value of ESR, CRP combined with ALB was 0.649. The AUC value of ALB, GLO combined with AGR was 0.709.

**Table 5:** Comparison of Serum Marker Levels between the Subgroup 1 and Subgroup 2 Patients before Reimplantation

Markers	Subgroup 1 (*n* = 11)	Subgroup 2 (*n* = 44)	*P* value
WBC (× 10^9^/L)	7.01 ± 1.69	6.93 ± 2.03	0.983
ESR (mm/hr)	27.46 ± 16.73	22.84 ± 18.40	0.328
CRP (mg/L)	9.82 ± 5.60	8.84 ± 12.56	0.090
ALB (g/L)	40.93 ± 3.45	43.40 ± 9.59	**0.041**
GLO (g/L)	33.55 ± 5.09	30.22 ± 3.45	0.088
AGR	1.27 ± 0.22	1.48 ± 0.32	0.067

Subgroup 1, reinfection; Subgroup 2, non-reinfection. *P* value was calculated by the Mann–Whitney U test. Bold numbers indicate a statistical difference

**Table 6:** The performance of single and combinative serum biomarkers in predicting reinfection following reimplantation

Serum markers	Sensitivity (%)	Specificity (%)	PPV (%)	NPV (%)	AUC (95% CI)	Threshold	Youden Index
WBC	27.27	52.27	12.49	74.19	0.502 (0.364 to 0.640)	7.3 × 10^9^/L	0.2045
ESR	100	22.73	24.45	100	0.596 (0.455 to 0.726)	7 mm/hr	0.2273
CRP	63.64	79.55	43.76	89.75	0.666 (0.526 to 0.788)	8.58 mg/L	0.4318
ALB	81.82	63.64	36.0028	93.3343	0.700 (0.562 to 0.816)	42.7 g/L	0.4545
GLO	63.64	70.45	34.9978	88.5718	0.667 (0.527 to 0.789)	32.5 g/L	0.3409
AGR	100.00	31.82	26.83	100	0.679 (0.539 to 0.798)	1.6 g/L	0.3182
ESR + CRP	100	27.27	25.58	100	0.605(0.464 to 0.735)	-	-
					0.649(0.508 to 0.773)		
ALB + ESR + CRP	72.73	61.36	31.99	90	0.709(0.571 to 0.823)	-	-
ALB + AGR + GLO	54.55	84.09	46.09	88.09		-	-

*ROC* Receiver operating characteristic, *PPV* Positive predictive value, *NPV* Negative predictive value, *AUC* Area under the curve, *WBC* White blood cell count, *ESR* Erythrocyte sedimentation rate, *CRP* C-reactive protein, *ALB* Albumin, *GLO* Globulin, *AGR* Albumin to globulin ratio

## Discussion

Accurate diagnosis of PJI is a precondition for successful treatment [17]. Although numerous valid biomarkers have been reported, such as α-defensin, leukocyte esterase, and interleukin-6 [18–20], they cannot be widely applied since most grassroots hospitals lack the resources to perform elaborate acquisition techniques and incur high detection costs. Consequently, there continues to be imperious demands for efficient, convenient, and inexpensive serum detection indicators for the diagnosis of PJI. Studies have demonstrated that serum protein levels change regularly in inflammatory and infectious diseases and have been used as auxiliary diagnostic markers for cancer, hepatitis, and other diseases by oncologists and gastroenterologists [10–12]. What is more, serum protein has the advantages of easy access and low cost. Hence, in this retrospective study, we verified that serum AGR, ALB, and GLO are promising and economical novel biomarkers for the diagnosis of PJI by evaluating 241 patients.

In a recent study, Ye et al. retrospectively analyzed the serum protein levels of 38 patients with PJI and 89 patients with aseptic loosening [13]. The levels of serum total protein and GLO in the PJI group were significantly higher than those in the aseptic loosening group, and the levels of ALB and AGR in the PJI group were significantly lower than those in the aseptic loosening group. The AUCs (sensitivity, specificity) of GLO and AGR were 0.77 (78.95%, 69.66%) and 0.779 (65.79%, 78.65%), respectively. In our study, AGR (0.851) indicated better diagnostic performance than that of Ye et al. In reality, AGR had the second highest AUC and was not significantly different from the best CRP (0.862) and the third highest ESR (0.845) (all *p* > 0.05). Additionally, unlike the results of Ye et al., ALB had an acceptable AUC (0.757). More amusingly, our results showed that the levels of all protein markers in the PJI group were significantly different from those in the aseptic loosening group, which was slightly different from that of Ye et al. We speculated that the reason for this situation was that the too small sample size included in Ye et al.'s study led to some confounding factors. In terms of the recent emergence of novel biomarkers, the diagnostic accuracy of biomarkers in each study varies. A retrospective study by Tejbir S et al. showed that the AUC of D-dimer was 0.724 for PJI [21]. In a review of 4939 revision cases, Taylor et al. reported that the ability of platelet count to distinguish between PJI and aseptic loosening (AUC = 0.660) was questionable, but overall diagnostic accuracy was improved when ESR and CRP were combined [22]. In the study of Qin et al. on the diagnosis of chronic PJI with IL6, the AUCs of serum IL6 and synovial IL6 were 0.973 and 0.945, respectively [23]. In comparison, although the serum protein in our study was not the best diagnostic marker at present, the AUC of ALB and GLO was > 0.7, and the AUC of AGR was > 0.8. Furthermore, ESR, CRP combined with AGR had the best diagnostic accuracy, which’s AUC value was up to 0.891. These indicated that serum AGR could be regarded as promising adjunct markers for the evaluation of PJI.

Currently, two-stage revision is deemed to be the most valid treatment for chronic PJI in East Asia and North America, but the postoperative reinfection rate is approximately 33% [24]. The high rate of reinfection was due to the inability to accurately determine whether the infection had been eradicated after the first stage of revision surgery (removal of the infected prosthesis and insertion of antibiotic-loaded spacer) and before reimplantation. Over the past few decades, joint surgeons have made many efforts to explore valuable biomarkers to determine the timing of reimplantation, but little progress seems to have been made. Several studies, including the synovial fluid percentage of polymorphonuclear, serum ESR, serum CRP, IL6, PET, could not predict the timing of reimplantation on account of poor accuracy [25–27]. A prospective study carried out by Kheir et al. revealed that the specificity of the LE test was 100% for predicting persistent infection before reimplantation, but the AUC was only 0.632 [28]. The latest evidence shows that the AUCs of D-dimer and plasma fibrinogen for predicting persistent infection before reimplantation are 0.565 and 0.773, respectively [29]. On the basis of our results, only ALB was statistically significant (*P* = 0.041) by comparing the levels of biomarkers in the reinfected group and the non-reinfected group, while there was no significant difference in other markers.

Among the novel serum protein markers, ALB (AUC = 0.700) had acceptable AUC values. In comparison to the above studies, the ALB of this study seems to be an available marker for predicting reinfection following reimplantation. The diagnostic accuracy of ALB, GLO combined with AGR is similar to that of ALB alone.

To the best of our knowledge, this study is currently the largest sample size to explore the value of serum AGR, ALB, and GLO in the diagnosis of PJI. In addition, this is the first study performed on the application of serum AGR, ALB, and GLO for reimplantation of two-stage revision arthroplasty. Admittedly, this study has certain limitations. First, even if the 2013 MSIS criterion is regarded as the gold standard in this study, several PJI patients may still be misdiagnosed with aseptic loosening. Second, this study is a single-center, single-ethnic study, and its usability in African or European populations is unknown. Finally, the baseline data of Group A and Group B were significantly different in terms of joint location, which may introduce some bias to the results.

## Conclusion

In our study**,** AGR, ALB, and GLO were all significantly correlated with PJI and can potentially be used as biomarkers to evaluate PJI. AGR was a promising adjunct marker for the diagnosis of PJI, similar to the classic markers CRP and ESR. ALB and GLO have acceptable values for the diagnosis of PJI. ALB may be expected to be an effective biomarker for predicting reinfection following reimplantation.

## Data Availability

The datasets used and/or analyzed during the current study are not publicly available due to feasibility but are available from the corresponding author on reasonable request.

## Abbreviations

PJI: Periprosthetic Joint Infection
TJA: Total Joint Replacement
ALB: Albumin
GLO: Globulin
AGR: Albumin to Globulin Ratio;
MSIS: Musculoskeletal Infection Society
WBC: White Blood Cell count
ESR: Erythrocyte sedimentation Rate, and
CRP: C-Reactive Protein;
ROC: Receiver Operating Characteristic
AUC: Area Under the Curve
PLR: Positive Likelihood Ratio
NLR: Negative Likelihood Ratio
